# Olfactory Virtual Reality: A New Frontier in the Treatment and Prevention of Posttraumatic Stress Disorder

**DOI:** 10.3390/brainsci11081070

**Published:** 2021-08-16

**Authors:** Rachel S. Herz

**Affiliations:** 1 Department of Psychiatry and Human Behavior, Brown University Medical School, Providence, RI 02912, USA; rachel_herz@brown.edu; 2 Department of Psychology and Neuroscience, Boston College, Chestnut Hill, MA 02467, USA

**Keywords:** PTSD, olfactory/odors, virtual reality, treatment, perception, emotion

## Abstract

This perspective piece reviews the clinical condition of posttraumatic stress disorder (PTSD), which is currently increasing due to the COVID-19 pandemic, and recent research illustrating how olfaction is being incorporated into virtual reality (VR) platforms. I then discuss the latest work examining the potential of olfactory virtual reality (OVR) for the treatment of PTSD. From this foundation I suggest novel ways in which OVR may be implemented in PTSD therapy and harnessed for preventing the development of PTSD. Perceptual and chemical features of olfaction that should be considered in OVR applications are also discussed.

## 1. Olfactory Virtual Reality: A New Frontier in the Treatment and Prevention of PTSD

The aim of this perspective piece is to discuss the potential for using new methodologies made possible through olfactory virtual reality (OVR) for the treatment and prevention of posttraumatic stress disorder (PTSD). Brief overviews of PTSD and virtual reality (VR) are first presented as a foundation, prior to delving into clinical applications of OVR for PTSD. For a comprehensive review of PTSD and olfaction [1]. For systematic reviews on the use and efficacy of VR treatment approaches for PTSD, the reader is directed to [2,3,4], among others.

## 2. Posttraumatic Stress Disorder

PTSD is a highly debilitating emotional disorder characterized by extremely distressing thoughts and intrusive traumatic memories. It is frequently accompanied by depression, substance abuse, sleep problems, and anxiety. In 2013, the American Psychiatric Association reported the following statistics for PTSD: it affects approximately 3.5 percent of U.S. adults; approximately eight million people are diagnosed with PTSD each year; and an estimated one in 11 people are afflicted with this condition during their lifetime [5]. Notably, since the COVID-19 pandemic began, alarmingly high rates of PTSD have been reported and a looming mental health crisis among healthcare workers and the general public has been declared [6,7,8,9,10]. Thus, a much higher proportion of the population is now vulnerable to PTSD than prior to 2020, and rates are expected to increase over at least the next several years, particularly among children [11].

PTSD can occur in anyone, although women are twice as likely as men to be diagnosed with this condition, and minorities in the United States, especially Latinos, African Americans, and Native Peoples, are disproportionately affected compared to non-Latino whites [5]. The nature of certain occupations, such as first responders and combat military personnel, also increases the risk of PTSD. The US Department of Veterans Affairs reports that up to 20 percent of veterans returning from the Iraq and Afghanistan wars are diagnosed with PTSD in a given year, and that the rate of PTSD for veterans of the Vietnam war is as high as 30 percent (https://www.ptsd.va.gov/understand/common/common_veterans.asp, accessed on 5 May 2021; see also [12,13]. Other individuals who are vulnerable to PTSD include survivors of shootings, bombings, violence, and rape. Family members of victims can also develop PTSD through vicarious trauma.

PTSD develops after someone has witnessed or experienced a grievous threat to life or physical harm, and is established through the mechanisms of fear conditioning. For example, sensory stimuli in the environment (sights, sounds, smells) that are present during the traumatic episode become associated with the emotions and experience of trauma such that subsequent exposure to any of these sensory cues evokes a highly traumatic and emotionally charged memory of the event, see [14] for review.

There are several trauma-focused treatments for PTSD that equip patients with strategies to confront and process traumatic memories and emotions, including cognitive processing therapy [15] and Eye Movement Desensitization and Reprocessing or EMDR [16]. The most widely used non-pharmacological treatment for PTSD is the cognitive behavioral technique of exposure therapy, in which the feared stimulus becomes muted through repeated exposure in a safe and supportive setting [17]. However, traditional exposure therapy has a number of limitations including, but not restricted to, access to stimuli, time constraints, and the inability to replicate certain situations. Moreover, success with general exposure therapy for PTSD is not always observed [18,19].

## 3. Virtual Reality

Virtual reality (VR) is a computer-simulated multidimensional environment in which the user wears a headset that eliminates most inputs from the external setting and creates new inputs within the inner surround. As the headset provides its own sensory and psychological landscape, it creates the experience of “presence”—the illusion of “being there” in a world that exists outside the self [20], where the external physical environment disappears from the user’s phenomenal awareness [21,22,23].

The condition of “presence” is the sought-after state in VR, and constitutes a complex psychological experience formed through the multifaceted interaction between sensory stimulation and resultant cognitive responses [24]. In addition, through a range of technology interface peripherals, such as hand controls or specialized interface devices, the individual is able to interact with the virtual environment and thus becomes “immersed” in the virtual space [25]. That is, immersion is achieved through technology, and presence is the psychological manifestation of being in a VR environment [26].

Two-dimensional VR has been available in the video gaming industry since the early 1990s. This basic VR is non-immersive—users interact with specialized interface devices and there is no occlusion of the surrounding environment [27]. By contrast, immersive VR aims to create a first person, three-dimensional (3-D) illusion of reality in which the external world is replaced by the virtual world [28]. The goal of immersive technology is to recreate the real world in all sensory domains; thus, all sensory attributes of reality must be mimicked [29,30]. Current work on the auditory component of VR has shown that spatialized sound (exploitation of sound localization through stereophonic headphones) augments the experience of 3-D space and increases presence, immersion, and VR task performance [29,31,32]. 

Until fairly recently, immersive VR applications were limited to visual and auditory stimulation and the omission of olfaction was seen as a shortcoming [33], for a review, see [26]. Olfaction is an especially important dimension to consider when creating an immersed facsimile of the real world; in addition to being a key component of lived experience, odors are particularly emotional and visceral stimuli [34]. Indeed, Munyan et al. [35] showed that including scent in VR environments increased the presence and stimulated real life scenarios more fully than VR without olfaction. Today, VR technologies are able to incorporate stimulation of all five senses [36]. With respect to including olfaction, there are various methods to deliver odors within VR, and the basic paradigm involves odor delivery and airstream technology integrated into the VR headset and a nosepiece system, see [37] for example. Possibilities for a spatialized 3-D olfactory environment are discussed in Section 6.2.

Because VR can create highly visceral and emotional experiences, and provide the facsimile of a lived encounter [23,38], it has become a very popular form of gaming and entertainment, and is increasingly being used in marketing [36], as well as psychological therapy [39,40].

In the clinical domain, VR is used predominantly and most effectively in affective conditions, such as PTSD [3]. Importantly, because VR can recreate almost any situation, and because it is highly emotionally involving, it is often used in exposure therapy, termed virtual reality exposure therapy (VRET). Recently, several comprehensive reviews and meta-analyses on the value of VRET have shown that VRET significantly reduces PTSD symptoms and is an efficacious treatment [3,4].

Of particular clinical significance for PTSD, odors may be the most incapacitating triggers [1,41,42]. This is because: (1) odors are invisible and there is often no way to prepare for or anticipate the possibility of exposure; (2) odors evoke more emotional and evocative memories than other sensory stimuli [43,44,45,46]; (3) odors are processed in the area of the brain where emotions, emotional memories, and associations are processed (the amygdala-hippocampal complex and orbitofrontal cortex), which comprises the same neural circuitry as PTSD [1,47]; (4) due to their direct connection to the neural network of emotion, odors can instantly elicit affective responses and associations prior to and without cognitive appraisal [48,49,50]. That is, at-risk individuals are vulnerable to traumatic odor-triggered memories; odor processing inherently elicits highly emotionally charged memories; and, due to the automaticity of odor-evoked memories, encountering an odor associated with trauma can sideswipe cognitively prepared coping responses.

In addition to being highly insidious triggers, odors often play a central role in PTSD episodes. For example, in a report of 100 refugees who were seen at a psychiatric clinic, 45 percent reported experiencing an odor-triggered panic attack within the preceding month [51]. Odor cues can also trigger particularly negative flashbacks. A laboratory investigation in healthy college students found that when memories for a disturbing film were examined one week after viewing, the odor associated with the film induced more arousing, detailed, and unpleasant memories than the associated auditory cue [52]. Thus, as has been remarked by researchers from diverse research perspectives, e.g., [1,53], the incorporation of odors into exposure therapy for PTSD is critically needed. Of note, a recent implementation showed that VRET with trauma-related odors (e.g., diesel fuel) in addition to visual and auditory stimuli was successful at reducing PTSD in military personnel [54]. This “BRAVEMIND” system, developed by Rizzo et al. [55] for combat-related PTSD therapy, is one of the most commonly used clinical VRET applications and can accommodate multisensory (e.g., olfactory) stimulus delivery. See Rizzo and Shilling [28] for a discussion.

The aim of the remainder of this article is to suggest several novel methods using OVR to aid in the recovery and mitigation of PTSD. The final sections address important factors in olfactory perception and OVR technology that should be considered to successfully implement these applications.

## 4. Implementation of OVR for PTSD Treatment

### 4.1. Systematic Desensitization with Olfactory Virtual Reality Exposure Therapy (OVRET)

Systematic desensitization is commonly used for the treatment of anxiety [56], and is similar to graded exposure therapy with the addition of muscle relaxation training. Systematic desensitization in prolonged exposure therapy involves beginning with a benign version of the traumatic trigger in a relaxation training setting and having the patient gradually work up to more faithful representations of the trauma-eliciting cue, such that they can eventually be exposed to the “real thing” while maintaining calm. By heightening emotional engagement, VRET has recently been shown to be successful at reducing physiological hyper-arousal to trauma-related stimuli in PTSD [57]. 

Importantly, as discussed previously, the neutralization of odor triggers is particularly relevant for treating PTSD. To implement systematic desensitization with olfaction and VR, odors that are similar to the odor associated with a trauma can be smelled during relaxation training, while the patient is guided through a virtual environment relevant to the trauma, until relaxation can be maintained. As treatment progresses, exposure to odors more precisely related to the offending odor can be presented until the person is able to relax fully in the presence of the trigger odor while simultaneously being exposed to the trauma-inducing scene in VR.

Notably, a problem for exposure therapy involving odors is that odor-associative learning is very specific [58,59]. For example, a memory triggered by the perfume *Joy* will not be triggered by the scent of *Chanel No. 5*. Thus, establishing the validity of a trauma-related odor cue with an olfactory facsimile is difficult, and the exact odor cue may be too overwhelming at first. Nonetheless, odors within a similar olfactory category can be used to ease patients into olfactory exposure therapy [60]. For instance, if the smell of diesel fuel triggers traumatic memories for a combat veteran, the odors of creosote, gasoline, and kerosene may be used in the initial phases of treatment. Gradually, the individual can then work up to being exposed to the trigger odor while maintaining calm during OVR simulations of the combat event. From personal communications with therapists working with PTSD patients to whom I have suggested relaxation training with graded olfactory stimuli, it is predicted that implementation of this strategy with OVR will effectively and more quickly defuse the trigger odor than using this general method in a non-VR setting.

### 4.2. Reappraisal of a Traumatic Memory with OVR

Emotional appraisal theory contends that how a person thinks about or “appraises” an event leads to traumatic emotions, rather than the event itself being the cause of emotional trauma [61]. Reappraisal is a cognitive strategy in which the individual learns to reframe the meaning of an event to be less distressing. For example, reappraisal of a video showing “disgusting” surgical procedures would involve instructions to take the perspective of a medical professional watching a training video and to focus on technical aspects of the operation [62]. Reappraisal strategies in general have been shown to increase emotional control and social functioning, and to improve psychological and physical well-being [63].

Reappraisal of an emotionally negative event to be less emotionally meaningful and intense has been shown to be effective at reducing PTSD symptoms [64,65]. For example, veterans with PTSD who were successfully trained to use reappraisal strategies and were self-aware of the emotions they were experiencing had lower PTSD symptom severity and greater positive affect [64].

To incorporate reappraisal training in OVR treatment for PTSD, olfactory counterconditioning can be implemented in which an odor associated with trauma is presented in an OVR environment and the person taught to re-appraise the meaning of the odor to be less emotionally negative. For example, if the aroma of grilled meat elicits an association of burning flesh, reappraisal OVR therapy could be implemented in several ways. One method would be the have the person repeatedly smell the odor of grilled meats in various pleasant BBQ and party scenarios in OVR, with instructions to think of the smell as only related to social get-togethers and backyard cookouts. Another method would be to have the person smell grilled meat while exposed to a combat scene, but told that the aroma comes from what is being cooked nearby and not from burning bodies. Theoretically, after such OVR reappraisal training, future encounters with the odor will have a more neutral meaning and any memories evoked will be less traumatic.

In addition to reducing self-reported symptoms of distress, re-appraisal has been shown to reduce amygdala activation [66]. Denny et al. [67] found that deliberate reappraisal increased responses in the prefrontal cortex, and decreased amygdala activity and self-reported emotion; moreover, these changes were long lasting. This suggests that reappraisal training can produce sustained alterations in the neural representation of an unpleasant event’s emotional value and increase emotional control. Importantly, the amygdala is the neural locus for the processing of olfaction, emotion, and emotional memory. Thus, it is expected that OVR reappraisal training can lead to a dampening of the neurological potency of olfactory cues, specific affective memories, and the patient’s emotional lability.

A limitation of olfactory reappraisal is that proactive interference is very strong in olfaction [68,69] and learning new associations with an odor, especially when the current emotional meaning is negative, is more difficult than with other sensory stimuli. However, the olfactory system is also extremely flexible and learning is the central feature of this plasticity [50,70,71,72]. It is thus predicted that, more than any other technique for learning new associations, the emotional vividness and multisensory features of OVR reappraisal training have the potential to establish new and benign associations for a trigger odor.

Evidence for the ability of odors to create particularly positive experiences in VR was recently shown in a study that demonstrated that odors increased the pleasantness of visual scenes in a virtual environment [73]; in another study, the presence of congruent odors augmented the desirability of and cravings for visual presentations of food in a virtual environment [74]. These examples illustrate the potency of odors to augment positive experiences in VR and their potential for therapeutic benefits.

### 4.3. Reducing Negative Affect with Odor Cues through OVR

It is well known that odors with positive emotional associations can be used to repair unpleasant emotional states and curb unhealthy behaviors [75,76]. For example, subjectively pleasant odors can improve mood [77], increase nostalgia and its psychological benefits [78], decrease cravings for cigarettes among smokers [79], and lower urges for unhealthy foods [80,81]. Furthermore, odors that elicit positive emotional states, elicit concordant downstream effects on physiology [48,82]. For example, an odor that induces calming emotions will also slow heart rate and respiration rate, and improve immune responses [83,84].

Based on these principles, positive emotional associations with odors can be used to reduce the unpleasantness of traumatic memories, irrespective of whether the traumatic memory is triggered by an odor; the trigger can be olfactory but also visual, auditory, or psychological. For instance, after OVR training with a soothing odor, whenever an individual is feeling emotionally overwhelmed by a traumatic recollection, they can sniff the calming odor to de-escalate their emotions and alter their mindset. Using OVR to establish relaxation with odors can thus lead to the reduction of a variety of unwanted states.

In addition to using odors that, for a given individual already possess positive associations, a “de-stress odor” can be created through OVR. Through similar procedures as both cue reappraisal and relaxation training with OVR, positive and peaceful emotional and physical associations can be conditioned to an odor that does not have any prior personal associations and tailored for the individual’s personal odor preferences, past experiences, and trauma history. That is, an unfamiliar odor paired with relaxation training and imagery will become a proxy for the feelings and associations of peacefulness and, when later smelled, elicit a calming state. Once a therapeutic association has been formed, the individual can take the odor with them and sniff (there are a variety of methods for encapsulating odors into portable/small/convenient formats) whenever they feel they may be about to experience panic or be overwhelmed by a traumatic memory. An added benefit of this method is that repeatedly smelling an odor that elicits positive associations when undergoing a traumatic recollection is an implicit form of reappraisal training—negative affect is replaced by positive associations, and over time the severity of the traumatic memories themselves should be reduced. However, two caveats to this technique are noted: (1) with repeated use, an emotionally positive odor could become associated to the traumatic memory, such that it becomes ineffective at reducing distress, or worse, elicits the traumatic memory itself [60]; (2) in comparison to utilizing mental–verbal strategies acquired through cognitive training, there is the inconvenience of always needing to physically carry a therapeutic odor on one’s person, because a traumatic memory could arise at any time.

## 5. Implementation of OVR for the Prevention of PTSD

Equally important as treating PTSD is the ability to increase resiliency and mitigate the initial development of PTSD. OVR can be harnessed to do this. I suggest two novel methods that can be implemented through OVR and used to mitigate the development of odor-evoked PTSD in people employed in professions at high risk for experiencing trauma.

### 5.1. Pre-Emptive Re-Appraisal with OVR

The Comprehensive Soldier Fitness program [85] aims to improve emotional coping skills and increase resilience in combat personnel by practicing psycho-educational and cognitive coping strategies, and, among other techniques, incorporates reappraisal to prepare service members for combat. Taking resiliency training further is the STress Resilience In Virtual Environments (STRIVE) project where, in preparation for deployment, service members engage in interactive VR combat scenarios to acquire resiliency and context-relevant emotional coping and reappraisal strategies [86]. Assessments of VR training for stress management to decrease negative affect in military personnel preparing for combat suggest that it is a promising tool [87].

To the best of this author’s knowledge, however, VR resiliency training has not yet incorporated odor cues into the VR environment. Doing so would be highly beneficial for a range of professions in which personnel face risks of developing PTSD due to the traumatic nature of their work and the intense olfactory cues that are often encountered. By way of illustration, nurses, rescue workers, and combat personnel may be exposed to the odors of bodily fluids and vehicle combustion in a VR setting while learning to reappraise the odors’ affective value through distancing techniques. Additionally, pre-emptive re-appraisal training for first responders such as police officers could help reduce lethal mistakes made in states of heightened emotional stress. For instance, odors of fast-food, sweat, and smoke that are likely to be present during hostile police–civilian interactions could be presented in OVR scenarios along with reappraisal training (e.g., construing the odors as connoting friendly gatherings) as a method for pre-emptive de-escalation and to assist in learning to maintain emotional control under duress.

Memory for odors and the memories elicited by odors are both very long lasting [49,88,89,90]. Therefore, using OVR to simulate hostile, combative, and medically traumatic events, and reappraising them to neutralize the negative emotions (e.g., disconnecting oneself from the imagined outcome or event; imagining the outcome or event to be neutral or pleasant) is hypothesized to have enduring effects for altering the meaning of olfactory triggers, and for decreasing subjective negative emotion and amygdala reactivity [67]. In sum, OVR resiliency training with odors that are predicted to be encountered in distressing situations can be presented in reappraisal scenarios, such that the emotional intensity of both the odors and the events are subjectively and neurologically diminished. Then, at the time of any future encounter, the odors involved will be less likely to become associated with the event or to subsequently trigger traumatic memories.

### 5.2. Inducing Adaptation and Habituation to Potential Trauma Odors

As discussed below in Section 6.1 (olfactory perception considerations), odor adaptation and habituation, in which the threshold level for odor detection substantially increases (resulting in minimal odor perception) after prolonged or repeated exposure, is a major feature of olfactory perception [91]. Although adaptation and habituation are problems for maintaining the effectiveness of repeatedly sniffed odors for emotional support, these processes may be beneficial for the prevention of traumatic associations to odors that are likely to be present during distressing events. That is, repeated exposure in VR to odors that are expected to be encountered in crisis scenarios, such as diesel fuel, burning hair, and bodily fluids, will produce habituation to these odors, such that they subsequently become barely detectable [91]. 

For example, repeatedly exposing novice military personnel to the odor of diesel fuel in OVR battlefield scenarios prior to their deployment would lead to habituation to the scent of diesel fuel such that it becomes minimally detectable. This training would also help inure recruits to the trauma of the scenario itself. That is, use of OVR to induce habituation will both lessen the perception of the odor and reduce the emotional distress of the event itself. Note that in order to maintain minimal detection of these odors and sustain emotional resiliency, it will be important to continue OVR training throughout a potential risk period so that habituation persists. An added benefit of this technique is that, because habituation will greatly diminish the ability to perceive the odors encountered during distressing events, these odors will be much less likely to become associated with a traumatic event in the first place, and therefore much less likely to be able to trigger a memory (e.g., PTSD) in the future.

Although it does not generally occur, a point of caution is the potential development of odor sensitization, in which repeated exposure to an odor lowers its detection threshold (increases sensitivity). Individuals with neurotic personality tendencies have been found to be both more susceptible to odor sensitization [92,93], and the development of PTSD [94]. The findings of Cortese et al. [95] and Wilkerson et al. [96] discussed in Section 6.1 also indicate that increased sensitivity to threat-relevant odors occurs in PTSD. Therefore, when using an odor habituation paradigm, it is important to monitor PTSD patients for odor sensitization to prevent maladaptive effects from arising.

## 6. Olfactory Factors in OVR and PTSD

Both the sense of smell and VR technologies possess unique characteristics and challenges for the successful implementation of OVR applications aimed at treating and preventing PTSD. Below I review some of the factors for each to consider.

### 6.1. Odor Perception Considerations

As mentioned above, odor adaptation and habituation, in which detection of an odor becomes very weak after continuous or repeated exposure, occurs very quickly and can last for weeks [91,97,98]. Odor adaptation is a physiological process and occurs when an odorant is continuously present and the receptors specific to its detection cease responding to it. The precise length of time for adaptation to occur varies as a function of both the individual [99] and the odorant [100], but it can occur in less than a minute [98].

Odor habituation is a cognitive phenomenon, which may also have physiological components. It occurs when one is exposed to the same odor on a daily basis and sensitivity to that odor dramatically diminishes [97,101]. For example, textile workers who were exposed to acetone daily exhibited acetone detection thresholds that were eight times higher than unexposed subjects [102]. Unlike receptor adaptation, which can be undone in a few minutes, cognitive habituation requires weeks to reverse, even for pungent trigeminal stimuli such as acetone [102,103]. 

The problems that adaptation and habitation present for OVR-based therapies are that repeated exposure to the same odors will quickly lead to them no longer being detectable and, thus, no longer therapeutically beneficial. To mitigate this issue, a set of odors can be conditioned to relaxing positive emotional states in OVR training, and then rotated through, such that no one odor is used for a sustained period. It would also be helpful to explain odor habitation to patients and counsel against over-sniffing for emotional support. Reducing habituation would enable odors that are used for relieving emotional distress to have the best potential for long-term efficacy. Note however that, as mentioned above, repeated exposure may also lead to problems associated with odor sensitization for PTSD patients.

Another important consideration for the use of OVR in PTSD therapy is that the condition of PTSD itself appears to alter odor processing. In several studies, Cortese and colleagues [95,96] found that veterans with PTSD exhibited elevated sensitivity to threat-related odors (e.g., burning rubber, burning hair, diesel fuel, and gunpowder) as measured both by self-reported and objective odor testing. However, sensitivity to odors that were not associated with trauma were reduced in PTSD patients, and grey matter volume in both piriform and orbitofrontal areas (primary and secondary olfactory cortex) was also found to be diminished [95,96]. Interestingly, these abnormal responses to odors may be specifically related to the current presence of PTSD and not just the experience of trauma. Croy et al. [104] found that individuals who had suffered child abuse did not currently show enhanced sensitivity or responses to odors, whereas PTSD patients displayed faster response times specifically to an unpleasant (though not trauma-specific) odor. In a further study, Croy et al. [105] found that olfactory bulb volume was decreased in adults who had been maltreated as children [105]. Therefore, both past and current trauma appear to alter the neurological representation of olfaction.

The key issue is that odor dependent variability in sensitivity and altered olfactory processing in individuals suffering from PTSD need to be taken into account. In practice, therefore, it may be best if odors used in OVR exposure therapy are presented at low concentrations. This may be especially helpful during the early stages of introducing a trigger odor during desensitization training, as it has been shown that the concentration of an odorant can alter both its perceptual and neurological processing [106]. On the other hand, when presenting benign odors in OVR training for evoking a calm and pleasant distraction, higher than average concentrations may be preferable.

Another important factor in odor perception is the high degree of individual variation in the subjective interpretation of an odor—irrespective of trauma history. Different people, as a function of their personal background, will have different emotional associations to various odors and thus different responses. As an example, Goel and Lao [107] found that the personal connotation of peppermint odor determined whether it would facilitate sleep; peppermint either increased or decreased sleep onset latency as a function of whether the participant perceived the scent as arousing or calming—and not in expected ways. More globally, different cultures have different interpretations of a scent due to the meaning of the scent in that culture [108,109]. Additionally, due to genetic variability in odor receptor expression [110], odors are perceived at different baseline intensities that can alter their qualitative connotation. Individual variability and response unpredictability to odors has also been recently demonstrated with EEG measures [111]. Thus, establishing a set of odors that are specifically designed for an individual will be the best method for therapeutic interventions, and a “one size fits all” approach should be avoided.

### 6.2. OVR Technology Considerations

There are several basic issues related to olfactory perception and odor chemistry that need to be addressed to ensure successful implementation of OVR technology, see [112] for a review of basic olfactory perception. The first of these is sensory detection speed. The sensation and perception of olfactory stimuli is much slower than in other sensory modalities. For example, first order neural detection of auditory stimuli occurs within 20 ms [113] and visual stimulus detection within 45 ms [114,115]. Somatosensory detection is more complex and varies as a function of the skin site and stimulus involved; nevertheless, detection is generally within the same order of magnitude as that of visual and auditory detection [116,117]. By contrast, primary odor detection takes up to 450 ms [118,119], and this time nearly doubles when the psychological perception of the stimulus is taken into account. It thus takes at least 10–20 times longer for odors to be perceptually processed compared to other sensory stimuli. Additionally, air flow rate and volume alter the speed at which odorants can reach the olfactory receptors, and vary under different environmental conditions. Thus, although multisensory synchrony is typically experienced between visual, auditory, and somatosensory stimuli, even though the neural registration of these stimuli is not perfectly synchronous [120] due to both air flow and particularly slow neural response times, the timing of odor exposures in VR will need to be carefully calibrated and coordinated with the other sensory stimuli presented to achieve the perception of multisensory integration.

Related to the speed of odor detection is the speed of odor clearance, which is both much slower and harder to control than the turning on and off of other stimuli. Rather than instantly vanishing when no longer presented, the dissipation of odor stimuli is slow and dependent on airflow, including air current speed, temperature, and humidity. Therefore, miniature fans, air temperature controllers, and other systems to ensure that odors “disappear” when corresponding sensory stimuli in other modalities change will be needed in the hardware for VR olfactory peripherals.

A further problem to consider is how to produce the spatialized perception of distance and movement between the VR user and an odor source, as has been achieved with sound [29]. In order to create this three-dimensionality, the intensity of the odor will need to be varied; lower concentration implies greater distance, and concentration increases incrementally as the odor source becomes more proximal (in either vertical or horizontal planes). Odor spatialization may be especially difficult to accurately achieve because the actual distance between the odor delivery system and the user’s nose is fixed in the peripheral device, and there is not enough space in the hardware to create stereophonic odor flow. One possibility may be to use a specialized cannula so that airflow to each of the nostrils can be precisely controlled and/or differently calibrated. Despite the inherent difficulties, any consideration of odor spatialization will improve the presence, immersiveness, and three-dimensionality of the VR environment.

A different fundamental issue concerns the chemical nature of odorants. Odors are lipophilic and stick to polymers, and most peripheral technology in VR is polymer based. This has been a central problem behind the failures of most odor dispersion inventions; for example, the use of “aroma-rama” in movie theaters [60]. Odorant adhesion to VR hardware is a particular issue if more than one odor is used in an OVR device. Odor mixing on the polymer surfaces will both contaminate any currently emitted odor and lead to constant exposure to novel odor mixtures. That is, over time, unwanted odor mixtures will accrue on the apparatus surfaces and residually remain, leading to the constant presence of complex odor mixtures. Additionally, and more importantly, the intended odor percepts will be contaminated by the presence of prior odors, such that the target odors are no longer perceived as expected, and are thus unable to elicit the desired responses. To resolve this issue, odor delivery systems and headset peripherals in contact with odors need to be frequently checked for contamination and should be non-polymer based. The best methodology would be to adopt the techniques used for manufacturing olfactometers that are used in fMRI research, see [121] for example and to construct any parts of the device that come in contact with odorants from fluorinated ethylene propylene (e.g., Teflon); odors do not stick to Teflon and it is a light, flexible, and diversely applicable material. In sum, proper delivery of odor stimuli within VR is complex and needs to be assiduously engineered. Of note, Ischer et al. [37] reported the development of a system for odor delivery in VR that addresses many of these concerns. Commercially available OVR devices that meet these requirements are also being developed.

## 7. Conclusions

Odors are common and debilitating triggers of PTSD, and VR technology with odors has recently been developed for the treatment of PTSD. Due to the uniquely emotional features of olfactory processing and odor-evoked memory, OVR is singularly positioned to be effectively used in the treatment of PTSD, as well as in relaxation training and positive emotional inductions to alleviate PTSD symptoms in general. Equally importantly, OVR can be used to increase resiliency and thwart the acquisition of odor-evoked PTSD. Example treatment strategies for PTSD with OVR include systematic desensitization, prolonged exposure, reappraisal strategies, and conditioning odors to be used for emotional calming. Methods for the prevention of PTSD in vulnerable groups include neutralizing potential odor triggers through pre-emptive habituation, and reappraisal training. Notably, the successful use of OVR for PTSD will need to confront several olfactory perception and technological challenges, including odor habituation and the lipophilic nature of odorants.

Emotional distress and PTSD ensuing from living through the COVID-19 pandemic are expected to be major mental health problems in the coming years. To address these needs, OVR treatment methods may be widely called upon for implementation in diverse settings. In addition to PTSD, the use of OVR to treat a variety of emotional problems is promising. For example, in a test for improving psycho-social wellbeing with VR, the presence of a pleasant odor reduced negative affect on various measures, including hostility, anxiety, and distress, significantly more so than when no odor was present [122].

There is a broad and promising clinical horizon for OVR technology and its application. It is hoped that this perspective piece provides a stepping stone to further innovation, research, and practice for the treatment and prevention of PTSD, and paves the way for valuable OVR treatments to be developed for many clinical conditions, as well as for the general improvement of quality of life.
